# On the Gastric Juice and Its Office in Digestion

**Published:** 1854-11

**Authors:** John C. Dalton

**Affiliations:** New York


On the Gastric Juice, and its office in Digestion. By John C. Dalton, Jr., New York.—Though the gastric juice has been more fully and perhaps more successfully studied than any other of the digestive fluids, there are still some contradictory statements with regard to it, even among authors of the first eminence. Since the admirable observations of Dr. Beaumont on the person of Alexis St. Martin, the secretion has been examined, on the lower animals, by Blondlot, Schwann, Wasmann, Bernard, Lehmann, and others, whose researches have, in most essential points, confirmed those of the American observer. During the past year, I have studied the gastric juice, at various times, by means of fistula, artificially established in the stomach of the dog, after the method of Blondlot and Bernard. The secretion was almost always obtained by feeding the animal with raw or cooked meat, after a twelve hours' fast, and collecting and filtering the fluid that ran from the fistula during the first half hour afterward. The statements of Dr. Beaumont with regard to the intermittent character of the secretion were always found to be correct. The stomach, in the intervals of digestion, contains only a colorless mucus, usually alkaline in reaction, though sometimes neutral. The acid fluid begins to run by drops in about five minutes after the introduction of food, and in ten to fifteen minutes it flows freely—occasionally in a small stream—from the orifice of the fistula. It then runs so fast that four or five ounces can be collected from a medium-sized dog in the course of half an hour. During the first five minutes of its flow it is sometimes colorless, but afterward it has a faint amber tinge, resembling that of pale urine or the serum of the blood.

A little confusion of ideas seems to have existed in the minds of some observers with regard to the best method of obtaining gastric juice in a state of purity. Several have thought this could be best accomplished by irritating the gastric mucous membrane with substances not liable to be attacked by the secretion; as, for example, glass rods, metallic sounds, &c. Dr. Beaumont appears to have sometimes succeeded in obtaining a tolerably large quantity of fluid by this means. I have myself, however, never been able to obtain a sufficient quantity of the secretion in the dog by any other means than the introduction of food. The stomach will often refuse to secrete a single drop of acid liquor, though irritated almost constantly for a quarter of an hour by a metallic catheter. Other observers, also, subsequent to Dr. Beaumont, are all agreed that only a very small quantity can usually be collected by such means; and yet they continue to recommend and employ the method, under an idea that the gastric juice so obtained will be "purer" than that procured in any other way. A moment's consideration, however, will show that the fluid which is secreted under the influence of so artificial and unnatural a stimulus cannot be at all relied on as representing the real gastric juice; and that the only proper method of obtaining this is to present to the stomach its natural stimulus, viz: the food which its secretion is intended to digest. The mistake, however, has been frequently made, and will undoubtedly account for some incorrect statements with regard to the specific gravity of the gastric juice, its non-coagulability by heat, &c. Frerichs, for example, in *Wagner's Encyclopædia of Physiology* (Art. *Digestion*), directs that, in order to obtain a "pure" gastric juice, the animals in whom gastric fistulæ have been established "should be fed, while fasting, with *indigestible substances*, *pebble-stones*, *pepper-corns*, *&c.*, and the fluid from the stomach then examined." A singular attempt, certainly, to excite a "normal" secretion by means of substances which are altogether foreign and unnatural to it, and with which it has no physiological relation. The truth is, these authors make a pure assumption when they regard the fluid so collected as more "normal" than that obtained by other means. They do not seem, either to attach sufficient importance to the fact that these irritants can at best produce only a very scanty supply of the secretion. Frerichs himself says he has only been able to obtain in this way from forty-five drops to two drachms and a quarter of "pure" gastric juice. This is not surprising, since he employed the method least adapted to excite its secretion. The secretion of true gastric juice can, in fact, be excited only by food, and by those particular kinds of food which are to be digested by it. Loaf-sugar, given to the dog, will be dissolved and disappear altogether from the stomach, as I have repeatedly found, without causing any appreciable amount of secretion. The same thing may be said of starch paste, when given alone. I have even seen tough and indigestible pieces of tendon, introduced through the fistula, expelled again spontaneously, one after the other, without causing the flow of a single drop of acid fluid; while cooked meat, introduced in the same way, produced immediately a free secretion. It is evident, then, that to procure normal gastric juice, *i. e.* the fluid normally secreted during the process of digestion, we must make use of its natural stimulants; and we cannot hope to obtain it in any other way.

There are only two possible sources of impurity for the gastric juice taken from the stomach after the introduction of ordinary food. One is an admixture of saliva. This, however, is generally regarded as of little importance, and, at any rate, it is not to be avoided by any of the methods proposed as substitutes. Saliva is even liable to be swallowed in the intervals of digestion, when no food whatever has been taken. The other source of impurity, and that which has been thought more important, is the danger that the fluid taken from the stomach, even at the commencement of digestion, may have already dissolved some of the elements of the food, and thus present to the experimenter a complicated solution, instead of pure gastric juice. This, however, is not the case. If the animal be fed upon firm pieces of fresh meat, containing little blood—particularly if the fluids have been previously coagulated by a short boiling—a sufficient length of time elapses before solution begins for the experimenter to obtain an abundant supply of the unaltered secretion. The two principal products of digestion, albumen and albuminose, may be readily detected if present; and experiment shows that they do not exist in the fluid obtained by this means. There is, then, really no objection to the employment of this method; which is, indeed, the only reasonable and physiological one.

Gastric juice, obtained as above, has the following properties: Its specific gravity is 1010; its acid reaction is always decided; its viscidity is so slight as to be hardly noticeable; it becomes turbid by boiling, and by an excess of alcohol; it is not troubled by nitric acid, nor by ferrocyanide of potassium.

Its *organic ingredient*, concerning which so much has been said is undoubtedly the most important of its constituents. Though it has never been separated in a perfectly pure state, its presence is easily demonstrable. It may be the altered mucus of the stomach, as Bernard supposes, or a mixture of various organic matters, as believed by others; but it is certainly a peculiar substance, found nowhere else in the body, and essential to the digestive properties of the gastric juice. There seems no good reason why we should not retain for it the name of *Pepsin*, first given to it by Schwann; always recollecting, for the present, that this name does not indicate its chemical character or origin, but only its physiological destination. This pepsin resembles albumen, as stated by Robin and Verdeil, in being coagulated by heat. Lehmann and Frerichs are certainly in error when they assert the contrary; an error into which they have perhaps been led by regarding as true gastric juice an unnatural fluid, obtained by the unnatural means alluded to above. The coagulum thrown down on boiling fresh gastric juice is not albumen, as Lehmann intimates, since it is not precipitated by either nitric acid or ferrocyanide of potassium; and after gastric juice has been boiled and filtered it has lost its digestive power, though its acid reaction remains unaltered.

With regard to the *mode of action* of gastric juice, it may be confidently stated that it acts only on the albuminoid substances, and articles of food containing them—as meat, eggs, bread, nutritious vegetables, &c. It is also true that these matters are mostly converted by the action of gastric juice into albuminose, *i. e.* a substance which is not coagulable by heat, or by nitric acid, but which is best precipitated by an excess of alcohol. If the different articles of food be digested in fresh gastric juice in test tubes, at a temperature of 100° Fahrenheit, it will be found that the digestible ingredients of bread are all taken up under the form of albuminose. If cheese be treated in the same way, its casein is altogether converted into albuminose, and dissolved, while its oleaginous matters are set free, and collect as a yellow layer on the surface of the solution. A very considerable portion of raw white of egg, however, dissolves in the gastric juice directly as albumen, and remains coagulable by heat. Soft boiled white of egg and raw meat are principally converted into albuminose, but at the same time a small proportion of albumen is also taken up unchanged.

The gastric juice *actually dissolves the articles of food.* This has been, through some mistake, recently denied by one observer, so far as regards muscular tissue; the fibres being supposed to be simply swollen and partly disintegrated, but not actually dissolved in the stomach. The contrary, however, can readily be seen, by digesting raw meat, cut into small pieces, with fresh gastric juice at 100° Fahrenheit for eight or nine hours. The filtered fluid, which is perfectly clear, but rather more deeply colored than at first, has increased in specific gravity from 1010 to 1020, or 1022; and, by the addition of six or eight times its volume of alcohol, becomes excessively turbid and opaque, like thin milk. It is true that all parts of the muscular tissue do not dissolve; and there remains constantly, at the bottom of the test tube in which the digestion has been performed, a pulverulent, brownish sediment, consisting of the undigested portions of the meat. This is the case, also, with boiled white of egg. While the digestion is going on, it is easy to see that as the general mass of the fragment becomes transparent and gradually liquifies, there remain, scattered through its substance, numerous minute opaque spots which are not acted on; and which, when set free by the solution of the rest produce an opalescence in the fluid, and collect slowly as a sediment at the bottom. Whether these refractory portions are afterwards dissolved by the intestinal fluids, or whether they are altogether indigestible, and destined to be discharged with the excrements, is at present uncertain.

The *quantity of albuminoid matter dissolved by the gastric juice* has been variously stated. The method which I have adopted to determine this point is the following: it was first ascertained that the fresh lean meat of the bullock's heart loses by desiccation 78 per cent, of its weight; 300 grains of such meat, cut into small pieces, were then digested for ten hours in ℥iss of gastric juice at 100° Fahrenheit, the mixture being gently agitated as often as every hour. The meat remaining undissolved was then collected on a previously weighed filter and evaporated to dryness. When perfectly dry, it weighed 55 grains. This represented, allowing for the loss by evaporation, 250 grains of the meat in its natural moist condition; 50 grains of meat were then dissolved by ℥iss of gastric juice, or a little over 30 grains per ℥j.

From these data, we can form some idea of the large quantity of gastric juice secreted during the process of digestion. One pound of raw meat is only a moderate meal for a medium-sized dog, and yet, to dissolve this quantity (supposing the while of it to be digested), no less than sixteen pints of gastric juice will be necessary. This quantity, or any approximation to it, would be altogether incredible if we did not recollect that the gastric juice, as soon as it has dissolved its quota of food *is immediately reabsorbed*, and enters the circulation with the alimentary substances which it holds in solution; so that a very large quantity of the secretion may be poured out during the digestion of a meal at an expense to the blood, at any one time, of only two or three ounces of fluid. The simplest investigation shows that the gastric juice does not accumulate in the stomach in any considerable quantity, to remain there until the solution of all the food has been accomplished, but that it is gradually secreted so long as any food remains undissolved; that portion which has been already digested being disposed of by reabsorption with its solvent fluid. There is, then, during digestion, a constant circulation of gastric juice from the vessels to the stomach, and from the stomach back again to the vessels. Or perhaps it would be more correct to say that it is only the watery portions of the juice, holding sometimes in solution the digested albumen and albuminose, that perform this circulation; while its acid and organic ingredients remain very possibly in the stomach, ready to act on a new quantity of food, as that which has been already digested is withdrawn by absorption. That this is really the case, is proved by the following facts. First, if a dog be killed some hours after taking a meal, there is never more than a very small quantity of fluid found in the stomach just sufficient to penetrate and smear over the half-digested pieces of meat; and, secondly, in the living animal, gastric juice, drawn from the stomach when digestion of meat has been going on for six hours, contains little or no more organic matter in solution than that extracted fifteen to thirty minutes after the introduction of food. It has evidently been freshly secreted; and, in order to obtain gastric juice saturated with alimentary matter, it must be artificially digested with food in test tubes, where this constant absorption and renovation cannot take place.

*The time necessary for the digestion of food* varies apparently in different species of animals. In the dog, one pound of lean uncooked meat is completely digested, and disappears from the stomach, only in from eight to nine hours. From experiments of M. Colin, of the Veterinary School at Alfort, it appears that the process is equally long in the cat; and also, probably, in all purely carnivorous animals. Dr. Beaumont, on the contrary, found that in Alexis St. Martin, meat and bread were digested in five, four, and three hours; and even, in some instances, in an hour, or an hour and a half. This difference is undoubtedly in part owing to the influence of mastication, which is so very imperfectly performed in the carnivora. It might be supposed that, having constant opportunity of obtaining gastric juice from the dog, and of studying its effects in artificial digestion, we might easily determine the relative digestibility of the different articles of food used by ourselves. This, however, is not the case. The readiness with which a substance dissolves in gastric juice is only one element of its digestibility in the stomach. This depends also on the readiness with which it excites the secretion, and stimulates the peristaltic movements of the organ, the ease with which it disintegrates, the amount of previous mastication, &c. For the human species, then, the digestibility of various kinds of food can be properly determined only by direct observations on the human stomach, like those of Dr. Beaumont; and it will probably be long before we shall have any others so valuable, in this respect, as his.

*Amylaceous substances are not digested in the stomach.* It is easy to ascertain that cooked starch, artificially digested for ten hours in gastric juice, at 100° Fahrenheit, still exhibits its characteristic reaction with iodine. Bernard also maintains that it is equally unaffected in the stomach. Some writers, however, have thought that the well-known property of saliva in converting starch into sugar, would be exerted even in the stomach during the process of ordinary digestion. Lehmann for example, states that "sugar may be detected, by the ordinary means, in the stomach of the animal (with gastric fistula) in ten to fifteen minutes after it has swallowed balls of starch, or after they have been introduced through the fistula." I am at a loss to understand the grounds of this assertion, as I have never met with similar results. Either cooked or raw starch, administered mixed with meat or introduced alone through the fistula (the animal will not eat pure starch) is easily recognizable by its reaction with iodine, ten, fifteen, and thirty minutes afterward. In forty-five minutes it is diminished in quantity, and in one hour has almost invariably disappeared; but no sugar is to be detected at any time. Outside the body, too, gastric juice entirely prevents the saccharine conversion of starch by saliva. A thin solution of boiled starch, mixed with an equal quantity of saliva, and kept at 100° Fahrenheit, is completely converted into sugar in ten or fifteen minutes; but if an equal quantity of fresh gastric juice be previously added to the mixture, there is no trace of sugar, even at the end of three hours, and the starch retains its usual properties. It must be acknowledged, therefore, that though saliva converts starch outside the body, when digested alone in test-tubes, yet it does not have this effect, so far as we can ascertain, during the natural process of digestion in the stomach.

Cane sugar is slowly converted by gastric juice, outside the body, into grape. Ten grains of cane sugar, dissolved in half an ounce of gastric juice, and kept at 100° Fahrenheit, give traces of glucose at the end of two hours, and in three hours the quantity of glucose is considerable. It cannot be shown, however, that the gastric juice exerts this effect on sugar during ordinary digestion. If cane sugar be given to the dog while digestion of meat is going on, it disappears in from two to three hours, without any glucose being detected. There is a singular peculiarity, however, in the action of cane sugar, introduced into the stomach of the dog while empty. If half an ounce of loaf sugar be given to the animal after a twelve hours' fast, when the stomach contains no food or gastric juice, it almost invariably produces an immediate reflux of intestinal fluids (among which the bile is readily recognized by its color), and these promptly convert a part of the sugar which has been swallowed, so that in fifteen to thirty minutes the fluids extracted from the stomach contain an abundance of glucose. This, however, is only temporary. In forty-five minutes to an hour the intestinal fluids cease to be present, and the glucose at the same time disappears. Unaltered cane sugar, however, still remains and continues present for two and a half to three hours, when it gradually disappears without any subsequent production of glucose. During all this time, as has been mentioned above, no gastric juice is secreted; the reaction of the stomach remaining constantly neutral or alkaline.

The above experiments render it probable that even when cane sugar is taken with other articles of food, it is not converted into glucose in the stomach. We cannot, however, be positively sure of this, owing to a circumstance not generally known, viz: that *the presence of gastric juice interferes with Trommer's test for grape sugar.* If a drop of honey be mixed with a drachm or two of gastric juice, on adding sulphate of copper and potass, the solution takes a rich purple tinge instead of its ordinary blue color, and, on boiling, changes to yellow—but no copper is deposited. A mixture of honey and gastric juice, in equal volumes even, does not reduce the copper unless it be previously diluted with water; in that case the copper is deposited as usual. I have not been able to ascertain upon what ingredient of the gastric juice this singular property depends. It is not its free acid, since, if previously neutralized by potass, it still behaves in the same way. It is not its organic ingredient, since, if this be separated by boiling and filtering, it continues to act as before. Even the addition of several times its volume of alcohol does not altogether prevent the interference. A considerable proportion of glucose may still be recognized, notwithstanding the presence of gastric juice, by the change of color from purple to yellow above mentioned, though there may be no precipitate; but a small proportion does not show itself in any way. One sixteenth of a drop of honey in one drachm of gastric juice produces no change of color that could be at all relied on as a test; while the same proportion of honey in water reduces the copper promptly and abundantly. We cannot, therefore, detect a moderate quantity of glucose in gastric juice by this means.

It is universally agreed that oleaginous substances are not attacked by gastric juice, but are simply set free from their union with other matters. Thus, if adipose tissue be digested artificially, the walls of the fat vesicles, with the intervening areolar tissue, are dissolved, and the oil collects as a distinct layer on the surface. The oily matter of cheese acts in the same way, as already noticed Boiled yolk of egg is digested in a similar manner. Its albuminoid ingredients are dissolved out, while the fats collect at the top of the solution, as a mass of yellowish semi-solid granules.—*Am. Jour. of Med. Sciences.*

